# Temporal Whole-Transcriptomic Analysis of Characterized *In Vitro* and *Ex Vivo* Primary Nasal Epithelia

**DOI:** 10.3389/fcell.2022.907511

**Published:** 2022-06-15

**Authors:** Jelmer Legebeke, Katie L. Horton, Claire L. Jackson, Janice Coles, Amanda Harris, Htoo A. Wai, John W. Holloway, Gabrielle Wheway, Diana Baralle, Jane S. Lucas

**Affiliations:** ^1^ School of Human Development and Health, Faculty of Medicine, University of Southampton, Southampton, United Kingdom; ^2^ Southampton NIHR Biomedical Research Centre, University Hospital Southampton NHS Foundation Trust, University of Southampton, Southampton, United Kingdom; ^3^ School of Clinical and Experimental Sciences, Faculty of Medicine, University of Southampton, Southampton, United Kingdom; ^4^ PCD Diagnostic Centre, University Hospital Southampton, Southampton, United Kingdom

**Keywords:** primary nasal epithelium, air-liquid interface culture, airway cilia, physiological analysis, whole transcriptome analysis

## Abstract

Air-liquid interface (ALI) cell culture of primary airway progenitors enables the differentiation and recapitulation of a pseudostratified epithelium *in vitro*, providing a highly useful tool for researching respiratory health and disease. Previous studies into gene expression in ALI-cultures compared to *ex vivo* nasal brushings have been limited in the number of time-points and/or the number of genes studied. In this study physiological and global transcriptomic changes were assessed in an extended *in vitro* 63-day human healthy nasal epithelium ALI-culture period and compared to *ex vivo* nasal brushing samples. *Ex vivo* nasal brushing samples formed distinct transcriptome clusters to *in vitro* ALI-cultured nasal epithelia, with from day 14 onwards ALI samples best matching the *ex vivo* samples. Immune response regulation genes were not expressed in the *in vitro* ALI-culture compared to the *ex vivo* nasal brushing samples, likely because the *in vitro* cultures lack an airway microbiome, lack airborne particles stimulation, or did not host an immune cell component. This highlights the need for more advanced co-cultures with immune cell representation to better reflect the physiological state. During the first week of ALI-culture genes related to metabolism and proliferation were increased. By the end of week 1 epithelial cell barrier function plateaued and multiciliated cell differentiation started, although widespread ciliation was not complete until day 28. These results highlight that time-points at which ALI-cultures are harvested for research studies needs to be carefully considered to suit the purpose of investigation (transcriptomic and/or functional analysis).

## Introduction

Air-liquid interface (ALI) cell culture of primary airway progenitors enables the differentiation and recapitulation of a pseudostratified epithelium *in vitro*, with basal, goblet and ciliated cell populations interacting in a physiological manner. A major advantage of the ALI-culture platform is the flexibility to investigate the differences between healthy donors and patients with airway diseases such as asthma (Thavagnanam et al., 2014), chronic obstructive pulmonary disease (COPD) (Lee et al., 2020), cystic fibrosis (de Courcey et al., 2012; Schögler et al., 2017) and primary ciliary dyskinesia (PCD) (Walker et al., 2017). This ALI-culture method is used to facilitate PCD diagnostic testing when secondary cell health issues caused by inflammation and infections confound initial test results (Hirst et al., 2014; Coles et al., 2020). ALI-cultures are also used as models for testing therapeutic drug delivery and microbial infection e.g. effect of drugs on ciliary activity (Ong et al., 2016); a transmembrane conductance regulator potentiator in cystic fibrosis (McGarry et al., 2017); nitric oxide donors and antibiotics on non-typeable *Haemophilus influenzae* infection of PCD epithelium (Collins et al., 2017; Walker et al., 2017); virus infection of asthma epithelium (Hackett et al., 2011); bacterial lipopolysaccharide stimulation in COPD (Comer et al., 2013); airway barrier function during bacterial infection (Blume et al., 2020); anti-viral responses (Blume et al., 2017); SARS-CoV-2 virus infection (Blume and Jackson et al., 2021); and the ability to genetically manipulate these cells in culture for studies of gene function (Chu et al., 2015; Rapiteanu et al., 2020).

The ALI-culture method involves undifferentiated airway epithelial cells being densely seeded onto a porous membrane filter insert placed within a culture well. Here, the cells are expanded in submerged liquid-liquid interface until confluent before removing apical surface liquid thereby exposing the basal cells to air. The basal cells increase their membrane barrier function and become polarized and columnar with their nutritional supply provided only from the basolateral compartment. Differentiation factors in the medium signals airway epithelial cell differentiation and ciliogenesis; cilia growth is noted microscopically from the end of week 1 and cultures are typically considered fully differentiated between weeks 3–4. Cilia coverage varies with time but also by the composition of the differentiation medium, ranging from 5% to 50% (Serafini and Michaelson, 1977; Walker et al., 2017; Coles et al., 2020). Whilst the *in vitro* conditions enable a pseudostratified ciliated airway epithelium that produces mucin, the *in vivo* condition and cellular interactions and responses are far more complex (influenced by factors such as underlying disease, host immune response, airway microbiome, nutrient availability or environmental factors).

Previous findings have shown transcriptomic differences not only between donors of *ex vivo* brushing samples, but also between *ex vivo* brushing samples and ALI-cultures, which present a more stable transcriptional profile at end-points of 2–3 and 6 weeks differentiation (Pezzulo et al., 2010; Dvorak et al., 2011; Ghosh et al., 2020). Recently, Bukowy-Bieryłło et al. (2022) presented temporal transcriptional and functional data of 14 targeted cilia genes up to 28 days of ALI-culture (nasal cells from healthy donors) using PneumaCult medium. Using a different approach, whereby we give an overview of the whole transcriptome specific to each time-point, we present temporal expressional transcriptomic changes in healthy nasal epithelial cell ALI-cultures that were differentiated in Pneumacult medium and maintained for 63 days. We have determined which biological pathways are significantly regulated over the ALI-culture process and performed functional analysis to enable us to explain these changes. By comparing the temporal gene expression changes of *in vitro* ALI-culture with *ex vivo* nasal epithelium samples, we determine the *in vitro* time-points that best recapitulate the *ex vivo* situation. These data can provide a basis for future *in vitro* study designs that utilize airway ALI-cultures.

## Methods

Nasal epithelia were harvested from *n* = 14 healthy donors to provide enough material for both differentiation and transcriptomic analysis. High-speed video microscopy analysis (HSVMA) and trans-epithelial electrical resistance (TEER; of membrane barrier function) were carried out on *n* = 3. Scanning electron microscopy (SEM) on *n* = 2, and immunofluorescence on *n* = 3. Transcriptomic analysis was carried out on *n* = 3 per *ex vivo* sample and *n* = 3 *in vitro* ALI-culture time-points.

### Collection and Culturing of Nasal Epithelial Cells

Under local and national R&D and ethical approval (Southampton and Southwest Hampshire Research Ethics Committee A: CHI395 07/Q1702/109) inferior turbinate epithelium was brush biopsied from each nostril using two 3 mm bronchoscopy cytology brushes (Conmed, United States) (as per Rubbo et al., 2019). Nasal epithelial cells were cultured and differentiated as described in detail by Coles et al., 2020. In brief, basal epithelial cells from each donor were expanded using PneumaCult-Ex Plus Medium (STEMCELL Technologies, Canada) supplemented with hydrocortisone (0.1%) (STEMCELL Technologies), initially in one well of a 12-well culture plate (Corning Life Sciences, United States) and then a T-25 cm^2^ flask (Corning Life Sciences). Finally, 50,000–70,000 basal cells were seeded per PureCol collagen-coated 0.33 cm^2^ transwell insert (0.4 µm pore diameter polyester membrane insert; Corning Life Sciences, United States). When a confluent monolayer was observed (1–3 days), cells were taken to an ALI by removing surface liquid and replacing basolateral medium with PneumaCult-ALI Medium (STEMCELL Technologies) supplemented with hydrocortisone (0.5%) and heparin (0.2%) (STEMCELL Technologies).

All plastics were pre-coated with 0.3 mg/ml PureCol collagen (CellSystems, Germany) and cells at 50%–70% confluence were passaged with 0.25% Trypsin-EDTA solution (Sigma). After trypsinization Hanks’ Balanced Salt Solution (HBSS) as used to dilute enzymic activity and all centrifugations to pellet cells were done at 400 × g (for 7 min at room temperature). All media were exchanged 3 times weekly and contained 1% penicillin (5000 U/mL)/streptomycin (5000 μg/ml) (Fisher Scientific, Hampton, NH, United States, #15070063) and 0.002% nystatin suspension (10,000 U/mL) (Thermo Fisher Scientific) and cells were cultured at 37°C with 5% CO_2_ and ∼100% relative humidity.

### ALI-Culture Physiological Testing

The apical surface of the cultures were assessed for motile cilia coverage *in situ.* To remove mucus and/or debris prior to imaging, surfaces were washed three times with 100 µL HBSS. ALI-cultures were visualized using an Olympus IX71 inverted microscope, encased in an environmental chamber heated to 37°C, with a 20× objective lens. HSVMA videos were captured at every second field of view across the midline of the transwell insert using a Photron FASTCAM MC2 at 500 frames/sec. The percentage of motile cilia coverage was estimated by analysing twelve HSVMA.cih videos per transwell insert with a Fast Fourier Transform algorithm (ImageJ plugin, P. Lackie, Southampton, United Kingdom) (Coles et al., 2020). The same three healthy volunteer donors and transwells were used longitudinally.

For immunofluorescent labelling, membranes were washed three times with 100 μL phosphate buffered saline (PBS) and fixed *in situ* with 4% formaldehyde for 20 min at room temperature before being stored at 5°C in PBS. Membranes were excised, washed three times in PBS-0.1% Triton X-100, blocked with 100 μL 5% marvel solution in PBS-1% Triton X-100 at room temperature for 1 hour and washed again three times in PBS-0.1% Triton X-100. Cells were incubated at room temperature for 1 hour with primary antibodies anti-α-tubulin (Mouse; Sigma-Aldrich, United States; 1:50), anti-MUC5AC (Rabbit; Sigma-Aldrich, United States; 1:25) or anti-E-cadherin (Mouse; Takara, Japan; 1:200) in PBS-0.1% Triton X-100. Membranes were washed three times in PBS-0.1% Triton X-100. AlexaFluor 594 anti-rabbit or AlexaFluor 488 anti-mouse (Life Technologies, United States; both1:500) in PBS-0.1% Triton X-100 were added at room temperature for 1 hour. After washing three times in PBS-0.1% Triton X-100, cells were counterstained for 10 min at room temperature with DAPI (Molecular Probes, Thermo Fisher Scientific, United States; 1:500) in PBS-0.1% Triton X-100, then washed three times in PBS. Membranes were mounted between two coverslips using Mowiol (Merck, United Kingdom) and imaged on a Leica SP8 inverted confocal microscope using a 63× glycerol immersion lens.

One hour before TEER measurements were taken (also refer to Coles et al., 2020), 200 µL PneumaCult ALI medium was added to the apical side and 600 µL to the basolateral side; and cell and no cell control wells were incubated at 37°C. Before each measurement the electrodes were sterilized in 70% ethanol and rinsed in medium. The mean of three resistance readings from each transwell were corrected for background and normalized to the surface area of the insert (expressed as Ω.cm^2^).

The primary SEM fixative solution of 3% glutaraldehyde in 0.1 M cacodylate buffer pH 7.2 was added to the apical and basal compartments of the inserts which were kept at room temperature for 20 min before being stored at 5°C. Within 5 weeks from the first fixation, the samples were washed twice for 10 min with buffer (0.1 M cacodylate at pH 7.2), then post fixative (1% osmium tetroxide in 0.1 M cacodylate buffer at pH 7.2) was added for 1 h at room temperature. Samples were washed twice in buffer before undergoing a series of 30, 50, 70 and 95% ethanol dehydration steps, each for 10 min. Absolute ethanol was added twice, each for 20 min. Samples were critical point dried using Balzers CPD 030 critical point dryer (BAL-TEC, Liechtenstein) then sputter coated with silver DAG using an E5100 sputter coater (Polaron, United Kingdom). Images were captured using a FEI Quanta 250 scanning electron microscope (FEI, Netherlands).

### RNA-Seq of Nasal Brushings and ALI-Cultures at Different Time-Points Obtained From Healthy Donors

RNA-seq analysis was undertaken for different *in vitro* ALI-culture time-points (days 1, 4, 8, 14, 21, 28 and 63; *n* = 3 samples per time-point) and *ex vivo* nasal epithelial brushing samples (*n* = 3) which were stored in RNA-later^®^. Collection and sequencing of RNA was approved by the Health Research Authority (IRAS 49685) and the University of Southampton Research Ethics Committee (ERGO 23056). The RNeasy Plus Mini kit (Qiagen, Germany) was used for RNA isolation. The cytology brushes stored in RNA-later^®^ were transferred into lysis buffer (RLT Plus buffer with 1% β-mercaptoethanol) and vortexed. Lysis buffer was added to the *in vitro* samples and the membrane insert was pipette tip-scraped. All lysates used for the subsequent RNA isolation steps according to manufactures instructions. RNA quality and concentration was measured using an RNA Nano chip on the Agilent Bioanalyzer 2100. Samples with total RNA RIN score >6.8 were taken forward for cDNA library preparation and sequencing. cDNA libraries were prepared using Ribo-Zero Magnetic Kit for rRNA depletion and NEBNext Ultra Directional RNA Library library prep kit. The sequencing design used was 150 base pair paired-end reads at a sequencing depth of 20 million (Novogene, United Kingdom). Library quality was assessed using a broad range DNA chip on the Agilent Bioanalyzer 2100. Library concentration was assessed using Qubit and qPCR. Libraries were pooled, and paired-end 150bp sequencing to a depth of 20M reads per fraction was performed on an Illumina HiSeq2500 (Novogene), quality control of the RNA-seq data was performed using FastQC (Andrews, 2010) (v0.11.9), RSeQC junction annotation and junction saturation (Wang et al., 2012) (v4.0.0), and Picard insert size, RnaSeqMetrics assignment, RnaSeqMetrics strand mapping and gene coverage (Broad Institute, 2019) (v2.8.3) (codes used can be found in the Supplementary Material). Sequence reads were aligned with STAR basic two-pass mode (Dobin et al., 2013) (v2.7.3a) using human GRCh build 38 (Schneider et al., 2017) and GENCODE v35 gene annotation (Harrow et al., 2012), and subsequently sorted and indexed with SamTools (Li et al., 2009) (v1.3.2). Gene counts were obtained with HTSeq (Anders et al., 2015) (v0.11.2) using GENCODE v35 gene annotation and the union mode. Transcript per million (TPM) values were calculated with a custom in-house script. MultiQC (Ewels et al., 2016) was used to combine and assess the quality of the individual output files obtained.

### Transcriptome Comparison Between *Ex Vivo* Nasal Brushing and *In Vitro* ALI-Culture Time-Points

The raw gene counts obtained with HTSeq were used as input for EdgeR (Robinson et al., 2010) (v3.30.3) with R (R Core Team, 2020) (v4.0.2) in RStudio (RStudio Team, 2020) (v1.3.959). Experimental groups were defined as three samples for each of the eight time-points; the RNA-later^®^ group and the seven ALI-culture time-points. Genes with low counts were removed with the “filterByExpr” command and the data were normalized with the Trimmed Mean of M-values (TMM) method. Counts per million were calculated and used for principal component analysis (PCA) with prcomp, part of the stats package (R Core Team, 2020) (v3.6.2), parameters used were scale = TRUE, and the PCA plot was generated with ggplot2 (Wickham, 2016) (v3.3.2). Heatmap analysis was performed with pheatmap (Kolde, 2019) (v1.0.12) using the ward. D2 clustering method and euclidean clustering distance measure for the columns. BioLayout (Theocharidis et al., 2009) (v3.4) was used for gene co-expression analysis using default settings and a correlation value of 0.95. Gene clusters were visually assessed for gene cluster expression differences between the experimental groups. Genes within a gene cluster were analyzed with ToppGene (Chen et al., 2009) gene list enrichment analysis using the default settings to determine the underlying Gene Ontology (GO) (Ashburner et al., 2000; Gene Ontology Consortium, 2021) biological process terms. Differentially expressed genes were identified with EdgeR. Two experimental groups were defined as three samples for the RNA-later^®^ group and 21 samples for all the seven ALI-culture time-points each consisting of three samples. Differentially expressed genes were identified using an exact test. The volcano plot was generated with ggplot2 using the thresholds FDR *p*-value <0.05 and a log fold change of >|1|.

### Expression Analysis of Proliferative, Deuterosomal and Multiciliated Gene Markers

TPM normalized gene counts were used to assess the expression of previously identified gene markers for proliferative, deuterosomal and multiciliated cells (Ruiz García et al., 2019) in the nasal epithelial cells stored in RNA-later^®^ and cultured at ALI. Expression plots were generated with ggplot2.

## Results

### Temporal Characterisation of *In Vitro* ALI-Cultures During Differentiation and Ciliogenesis

**FIGURE 1:**
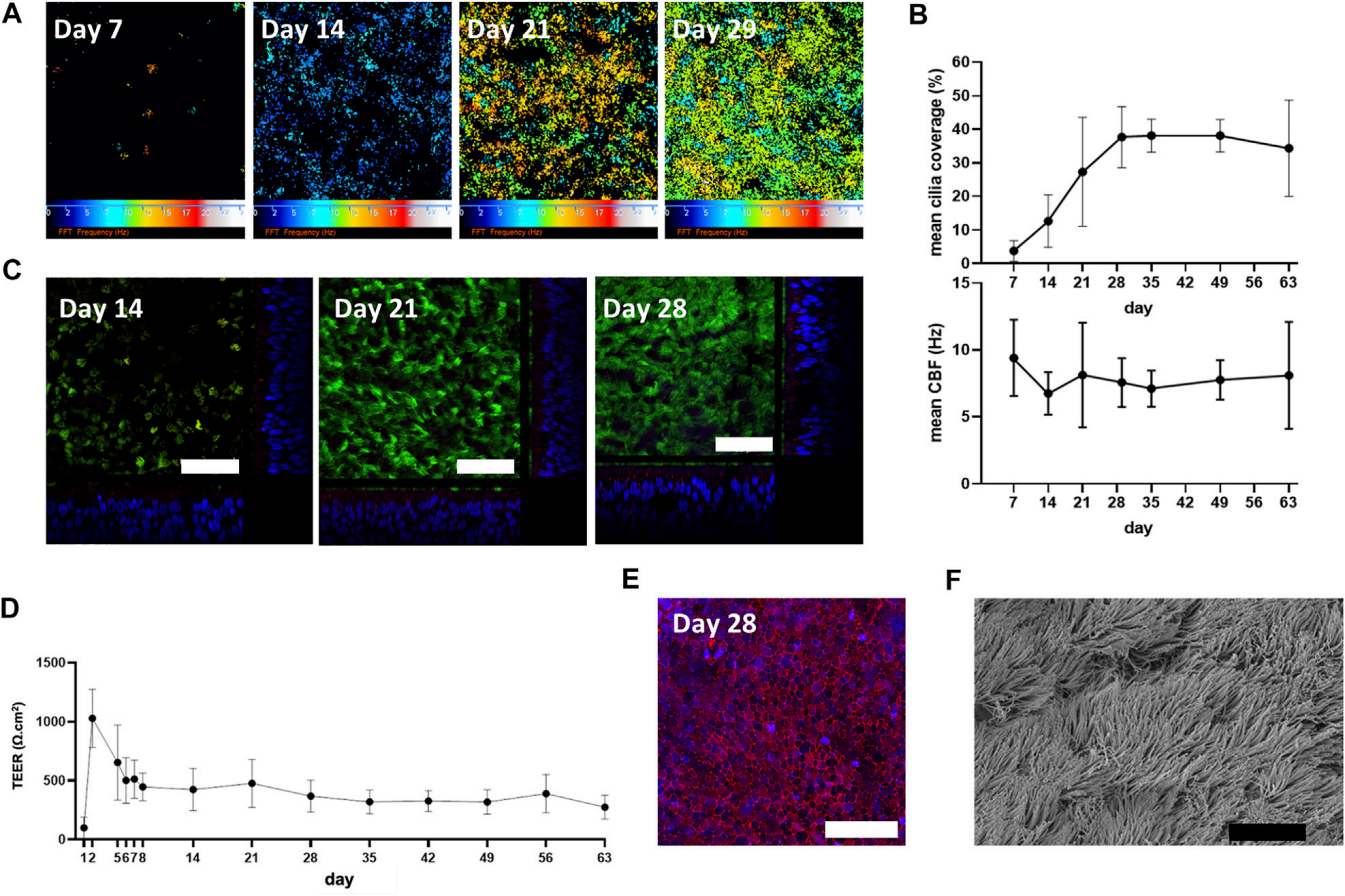
Physiological characterization of *in vitro* ALI-cultures. **(A)** “Colour map” outputs of ciliary movement detected by *in situ* high-speed video microscopy at 37°C. Colour scale represents increasing ciliary beat frequency (CBF) from 0 (black) to 25 Hz (white), where black also represents CBF measurements outside of the detection threshold (below 2 Hz or above 50 Hz). **(B)** Cilia were detected at day 7 with a weekly increase in percentage cilia coverage up to day 29 (plateau). Mean CBF (*n* = 3) was measured *in situ* at 37°C. **(C)** Immunofluorescence staining of a-tubulin (cilia; Alexafluor488 secondary antibody, green), MUC5AC (goblet cells; Alexafluor594 secondary antibody, red) and DAPI (blue) (representative images from *n* = 3). Orthogonal views show cellular position of α-tubulin and increasing cytoplasmic MUC5AC expression and epithelium height from day 7 to day 28. **(D)** Membrane barrier function was assessed by transepithelial electrical resistance (TEER) measurements with stability observed from day 28. Mean ± SD from *n* = 3. **(E)** Maximum projection shows total E-cadherin (cell-cell adhesion molecule; Alexafluor488 secondary antibody, red) and DAPI (blue) expression at day 28 (representative image from *n* = 3). **(F)** Scanning electron microscopy (representative of *n* = 2) supports widespread ciliation at day 28. White scale bar = 50 µm. Black scale bar = 10 µm.

The differentiation and ciliogenesis of *in vitro* ALI-cultures were assessed at weekly time-points (7, 14, 21, 29, 35, 49 and 63 days) by HSVMA to estimate cilia coverage and measure ciliary beat frequency (CBF). End-point immunofluorescence and SEM imaging were performed to confirm presence of differentiation markers and cilia integrity. HSVMA using *post-hoc* Fast Fourier transform analysis confirmed ciliary beating on *n* = 3 healthy donors. Cilia were detected at day 7 (mean cilia coverage = 4%, SD ± 3) and coverage increased weekly (13%, SD ± 8 and 27%, SD ± 16 on day 14 and 21, respectively) until reaching a plateau on day 29 (38%, SD ± 9). Cilia development remained stable on day 35 (38%, SD ± 5) with tight error bars so coverage was then assessed with two weekly measurements until day 63. At day 63, one culture was unmeasurable due to excess mucus. CBF was measured *in situ* at 37°C with minimal differences detected between timepoints (mean CBF remained between 7.1 and 9.4 Hz (Figures 1A,B)). Cilia production and cell differentiation were further characterized by immunofluorescent labelling. Staining started at day 14 because few cilia were detectable by HSVMA on day 7. Incremental cilia coverage and mucin production were demonstrated by increasing expression of cilia specific tubulin and intracellular MUC5AC labelling between days 14 and 28 (*n* = 3). Orthogonal views show the cellular positions of tubulin (apical surface) and MUC5AC (intracellular) (Figure 1C). As ciliation, determined by HSVMA, remained stable from week 4 onwards, day 28 was selected for SEM and confirmed widespread ciliation (*n* = 2) (Figure 1F). Secondary only antibody controls showed no non-specific binding (data not shown).

To assess the ALI-culture membrane barrier function over time, TEER was measured at days 1–2, 5–8, 14, 21, 28, 35, 42, 49, 56, and 63 (*n* = 3). A maximum mean TEER value of 1030 (SD ± 249) Ω.cm^2^ was observed on day 2. By day 5 mean TEER was markedly decreased [655 (SD ± 319) Ω.cm^2^] and gradually declined until day 8 (446 (SD ± 117) Ω.cm^2^) and then remained relatively constant until day 63 [274 (SD ± 100) Ω.cm^2^] (Figure 1D). Consistent with the formation of a polarized epithelial barrier, a maximum projection confocal image showed total E-cadherin labelling at day 28 (when TEER had plateaued), verifying tight junction formation (Figure 1E).

### Comparisons of Transcriptomes of *Ex Vivo* Nasal Brushing Samples and *In Vitro* ALI-Cultures, and of *In Vitro* ALI-Culture at Seven Time-Points

**FIGURE 2:**
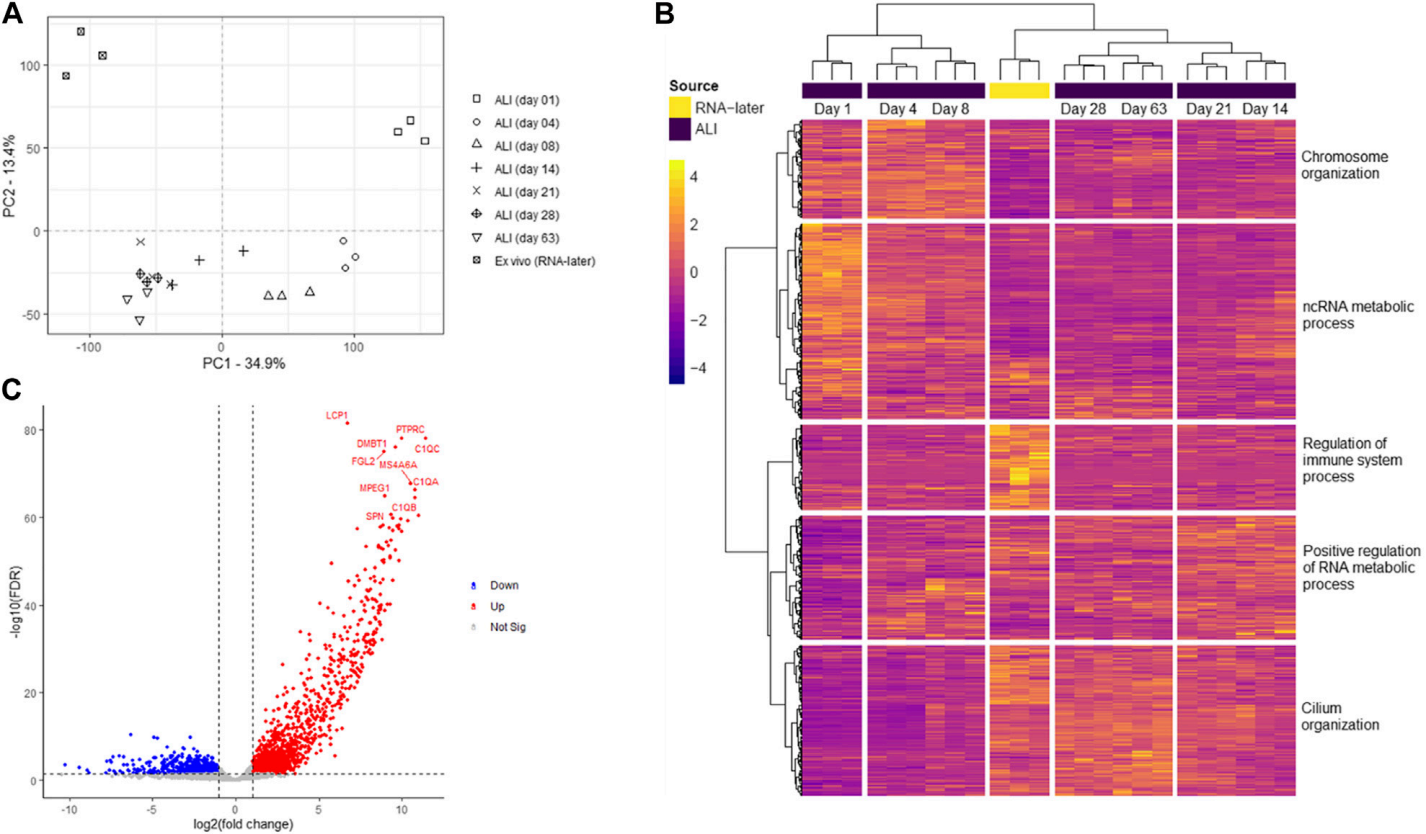
Transcriptome analysis of *ex vivo* nasal epithelial cells and *in vitro* ALI-cultures harvested at different time-points during differentiation and ciliogenesis. *Ex vivo* nasal brushing transcriptomes (RNA-later^®^) were compared to *in vitro* ALI-culture transcriptomes harvested at days 1, 4, 8, 14, 21, 28, and 63. **(A)** Principal component analysis (PCA) revealed that the RNA-later^®^ transcriptomes form a distinct separate cluster compared to the ALI-culture time-point transcriptomes. The ALI-culture day 1 transcriptome clusters separately to the other ALI-culture time-points. ALI-culture days 4 and 8 display a higher transcriptome similarity to each other compared to the other ALI-culture time-points, and the transcriptomes from day 14 onwards increase in similarity. Furthermore, the RNA-later^®^ transcriptomes appear to be most similar to ALI-cultures from day 14 onwards. **(B)** While heatmap analysis depicts similar transcriptome clustering as PCA further clustering was detected with ALI-culture days 14 and 21, and days 28 and 63, clustering together. Five major gene clusters with higher to lower expression are shown segmented from top to bottom: i) 3,023 genes, ii) 6,040 genes, iii) 2,626 genes, iv) 3,832 genes and v) 4,661 genes. The most statistically significant Gene Ontology biological process terms for each of the major gene clusters were: i) “chromosome organization” (FDR *p*-value 1.29 × 10^–57^), ii) “ncRNA metabolic process” (FDR *p*-value 9.96 × 10^–26^), iii) “regulation of immune system process” (FDR *p*-value 9.98 × 10^–41^), iv) “positive regulation of RNA metabolic process” (FDR *p*-value 1.86 × 10^–11^) and v) “cilium organization” (FDR *p*-value 1.04 × 10^–68^). **(C)** Volcano plot showing the ten most significantly upregulated genes in the *ex vivo* nasal brushing samples compared against all the seven *in vitro* ALI-culture time-points. Thresholds are FDR *p*-value <0.05 and a log fold change of >|1|. Three healthy donors were used for each time-point.

Transcriptomes consisting of 20,182 genes, of *ex vivo* nasal brushings stored in RNA-later^®^ (further referred to as *ex vivo* samples) and *in vitro* ALI-cultures harvested at seven time-points (day 1, 4, 8, 14, 21, 28, and 63), were compared by PCA analysis. The *ex vivo* samples formed a distinct transcriptome cluster, separate from any of the ALI-culture time-points. Furthermore, transcriptomic changes during ALI-culture cell differentiation and ciliogenesis resulted in further separate gene expression clusters at different time-points. Day 1 ALI-cultures, which contain unpolarized and undifferentiated basal epithelial cells, formed a separate transcriptome cluster to any of the later ALI-culture time-points. Day 4 and day 8 ALI-culture clusters were transcriptionally most similar to each other, while the transcriptome differences between timepoints from day 14 onwards appear less prominent (Figure 2A).

### Identification of Gene Cluster Expression at Different ALI-Culture Time-Points Indicate Changing Biological Processes

**FIGURE 3:**
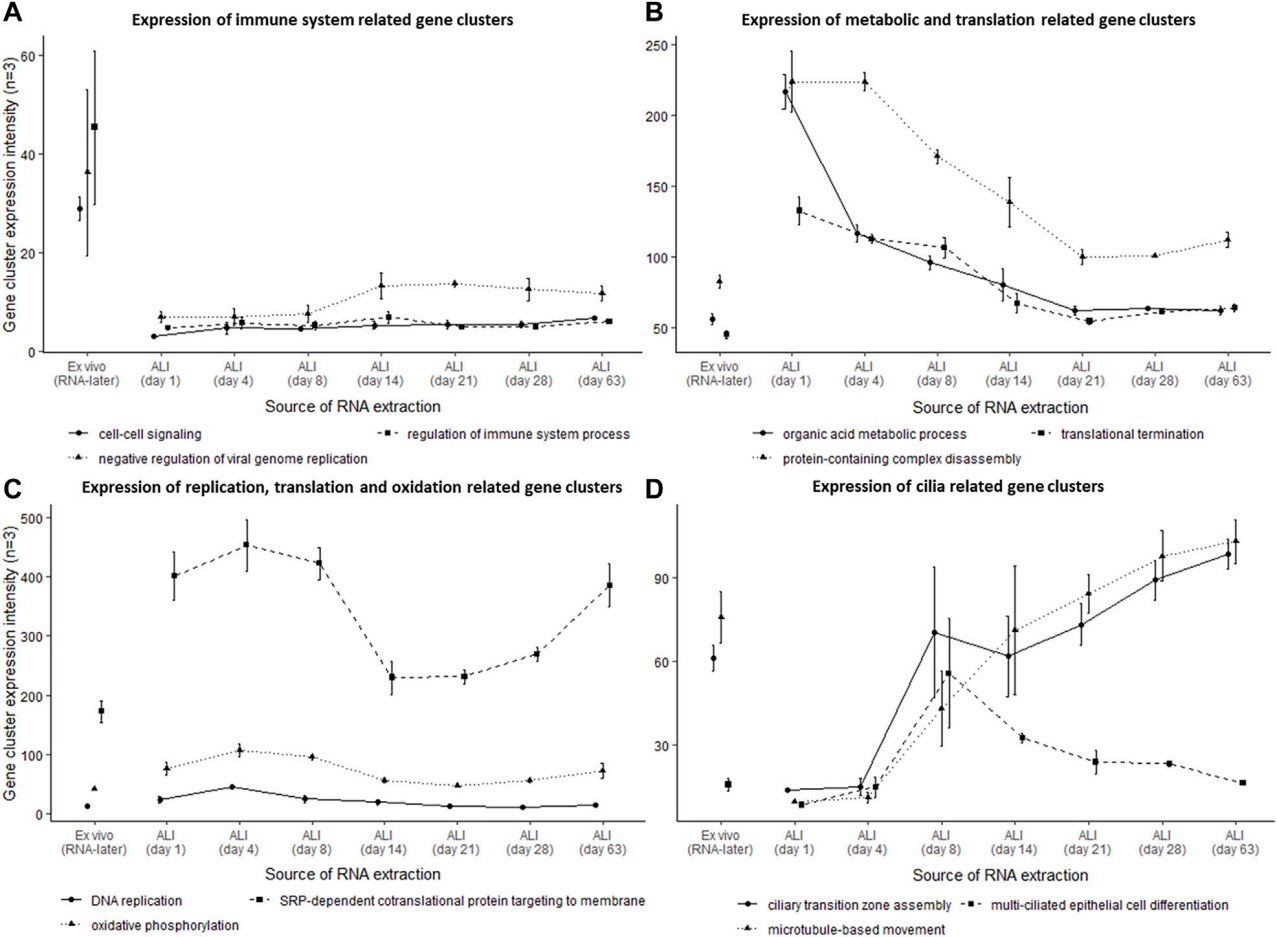
Temporal changes of distinct gene clusters associated with a wide range of biological processes. Gene co-expression identified temporal changes of several distinct gene clusters. **(A)** Gene clusters associated with the “regulation of immune process response” (FDR *p*-value 1.59 × 10^-63^), “cell-cell signalling” (FDR *p*-value 2.52 × 10^-03^) and negative regulation of viral genome replication” (FDR *p*-value 9.61 × 10^-03^) being substantially higher in the *ex vivo* nasal epithelial cells stored in RNA-later®. **(B)** While several gene clusters associated with “organic acid metabolic processes” (FDR *p*-value 17.96 × 10^-12^), “translational termination” (FDR *p*-value 4.62 × 10^-05^) and “protein-containing complex disassembly” (FDR *p*-value 2.60 × 10^-04^) were more prominently expressed at in vitro ALI-culture day 1, with the expression declining in the subsequent ALI-culture time-points. **(C)** Gene clusters associated with “DNA replication” (FDR *p*-value 2.52 × 10^-38^), “SRP-dependent cotranslational protein targeting to membrane” (FDR *p*-value 3.28 × 10^-29^) and “oxidative phosphorylation” (FDR *p*-value 2.47 × 10^-09^) were highly expressed at ALI-culture day 4, and appeared to become cyclic over the subsequent ALI-culture time-points. **(D)** Finally, a gene cluster involved “multi-ciliated epithelial cell differentiation” (FDR *p*-value 1.28 × 10^-04^) was highly expressed at ALI-culture day 8, and other gene clusters involved with ciliogenesis being “ciliary transition zone assembly” (FDR *p*-value 2.74 × 10^-09^) and “microtubule-based movement” (FDR *p*-value 1.31 × 10^-105^) were being substantially expressed from ALI-culture day 8 onwards. Three healthy donors were used for each time-point.

Heatmap analysis of the aforementioned transcriptomes confirmed a similar sample clustering to the PCA analysis as can be seen in the time-point dendrogram (Figure 2B). The PCA plot demonstrated transcriptomes from each sub-grouping e.g., the *n* = 3 *ex vivo* donors and the seven *in vitro* ALI-culture time-points (*n* = 3 samples per time-point), and these appeared to have low inter-donor variability. We measured the Biological Coefficient of Variation (BCV) between samples within each sub-grouping in edgeR and found the BCV ranged between 19% and 31%. A BCV between 20% and 40% is considered acceptably low variability to enable detection of differentially expressed genes. Undifferentiated basal cells on day 1 of ALI-culture formed a separate cluster unlike the *ex vivo* samples or the *in vitro* ALI-culture day 4 to day 63 time-points (during differentiation and ciliogenesis). ALI-culture days 4 and 8 (early basal epithelial cell polarisation and differentiation) formed an overlapping cluster. ALI-culture day 14 clustered with day 21 (differentiation and ciliogenesis peak), and days 28 and 63 forming another overarching cluster. Furthermore, the time-point dendrogram revealed that the *ex vivo* samples were most similar to the *in vitro* nasal epithelial cell ALI-cultures from day 14 onwards but closest to the overarching cluster representing days 28 and 63. Heatmap and gene co-expression analysis identified that genes with different biological pathways (gene clusters) were differentially expressed at different time-points (Table 1). The gene clusters specific to the *ex vivo* samples were associated with the “regulation of immune system process” (Figure 2B), “negative regulation of viral genome replication” and “cell-cell signalling” (Figure 3A). The mean TPM of the genes within the “regulation of immune system process” cluster was 30 in the *ex vivo* samples, while in the *in vitro* ALI-culture time-points the mean TPM fluctuated from 3 to 7. The ten top upregulated genes in the *ex vivo* samples, compared against all the seven *in vitro* ALI-culture time-points, are *LCP1*, *C1QC, PTPRC, DMBT1, FGL2, MS4A6A, C1QA, MPEG1, C1QB* and *SPN* (Figure 2C). On day 1 of ALI-culture, gene clusters were associated with “ncRNA metabolic process” (Figure 2B), “organic acid metabolic process”, “protein-containing complex disassembly”, “translational termination” and “ribonucleoprotein complex biogenesis” (Figure 3B). The expression of these gene clusters peaked on ALI-culture day 1 before decreasing throughout the subsequent ALI-culture time-points and being less expressed in the *ex vivo* samples (Figure 3B). On day 4 of ALI-culture, gene clusters were associated with “chromosome organization” (Figure 2B), “DNA replication”, “SRP-dependent co-translational protein targeting to membrane” and “oxidative phosphorylation” which after peaking on day 4 became cyclic in expression (Figure 3C). On day 8 there was a peak in expression of “multi-ciliated epithelial cell differentiation” (Figure 3D). The gene cluster associated with ALI-culture days 14 and 21 was “positive regulation of RNA metabolic process” (Figure 2B). The gene cluster “cilium organization” was associated with ALI-culture days 28 and 63 (Figure 2B). Genes associated with “microtubule-based movement” and “ciliary transition zone assembly” started to be expressed between day 4 and day 8 of culture, and this increased over the subsequent ALI-culture time-points (Figure 3D).

**TABLE 1:** Gene clusters identified by heatmap and gene co-expression analysis. ToppGene enrichment determined the underlying Gene Ontology (GO) biological processes across *ex vivo* nasal brushing samples (*n* = 3 healthy donors in RNA-later^®^) and *in vitro* ALI-cultures harvested at time-points during differentiation and ciliogenesis for 63 days (*n* = 3 healthy donors per time-point). Both analyses depict the same, with immune regulation related gene clusters associated with the *ex vivo* nasal epithelial cells in RNA-later^®^, metabolic processes with ALI day 1, chromosome organization with ALI day 4, differentiation of the nasal epithelial cells into multi-ciliated cells at ALI day 8, and primarily other cilia related gene clusters from ALI day 14 onwards.

Sample Group	Heatmap analysis		Gene co-expression analysis	
	GO biological process	FDR	GO biological process	FDR
*Ex vivo* (RNA-later^®^)	Regulation of immune system process	9.98 × 10^–41^	Regulation of immune system process	1.59 × 10^–63^
			Negative regulation of viral genome replication	9.61 × 10^–03^
			Cell-cell signalling	2.52 × 10^–03^
ALI (day 1)	ncRNA metabolic process	9.96 × 10^–26^	Organic acid metabolic process	7.96 × 10^–12^
			Translational termination	4.62 × 10^–05^
			Protein-containing complex disassembly	2.60 × 10^–04^
			Ribonucleoprotein complex biogenesis	1.09 × 10^–04^
ALI (day 4)	Chromosome organization	1.29 × 10^–57^	Chromosome organization	1.45 × 10^–74^
			DNA replication	2.52 × 10^–38^
			SRP-dependent cotranslational protein targeting to membrane	3.28 × 10^–29^
			Oxidative phosphorylation	2.47 × 10^–09^
ALI (day 8)			Multi-ciliated epithelial cell differentiation	1.28 × 10^–04^
ALI (day 14)	Positive regulation of RNA metabolic process	1.86 × 10^–11^	Microtubule-based movement Ciliary transition zone assembly	1.31 × 10^–105^, 2.74 × 10^–09^
ALI (day 21)				
ALI (day 28)	Cilium organization	1.04 × 10^–68^		
ALI (day 63)				

### Expression of Gene Markers Indicated Cell Type Changes at Different ALI-Culture Time-Points

**FIGURE 4:**
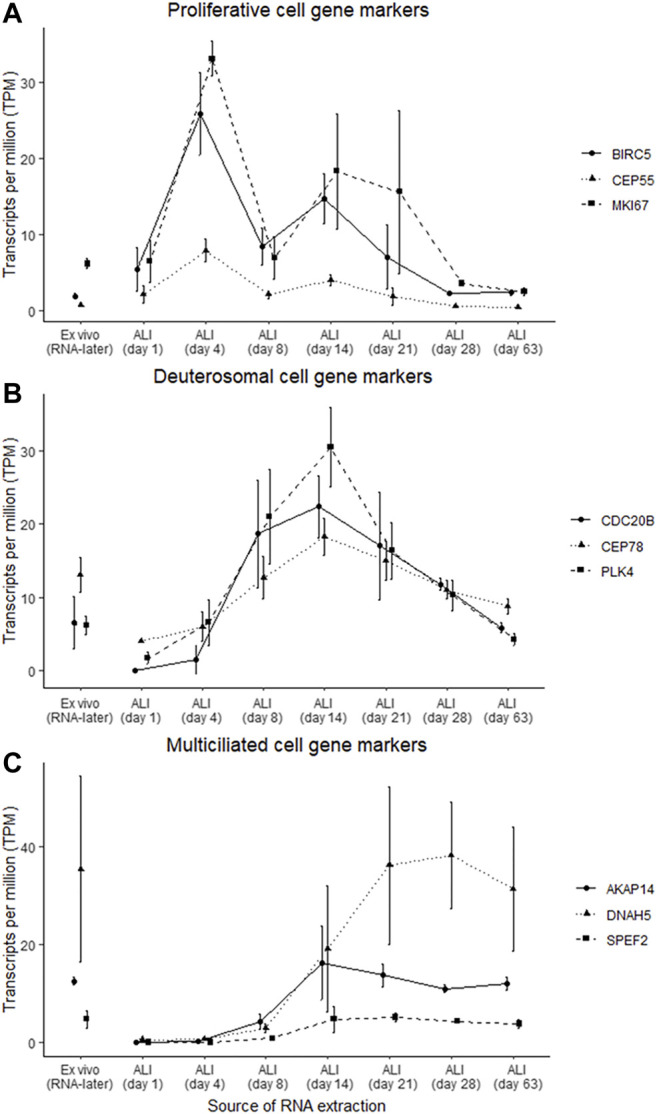
Gene marker expression changes indicated specific cell type changes at different ALI-culture time-points. Gene markers, which were previously identified (Ruiz García et al., 2019), belonging to proliferative, deuterosomal and multiciliated cells were assessed in the ex vivo nasal brushing cells stored in RNA-later® samples and the *in vitro* nasal epithelial cells cultured at ALI. **(A)** Peak abundance of the gene markers *(BIRC5, CEP55, and MKI67)* associated with proliferative cells occurs at day 4 of the ALI-culture. **(B)** Abundance of the gene markers *(CDC20B, CEP78, and PLK4)* associated with deuterosomal cells starts at ALI day 4 and peaks around day 14, while the gene marker *(AKAP14, DNAH5, and SPEF2)* transcripts of multiciliated cells appear from ALI day 8 onwards and peaking around day 14 and day 21. Proliferative and deuterosomal gene marker transcripts are more abundant in the ALI-cultures compared to the RNA-later® samples, while the abundance of multiciliated gene marker transcripts between the ALI-cultures and the RNA-later® samples are at similar levels around ALI day 14 and day 21 **(C)**. Three healthy donors were used for each time-point.

Gene markers for proliferative, deuterosomal, and multiciliated cells previously identified by single-cell RNA-seq of nasal epithelial cultures (Ruiz García et al., 2019) were used to assess specific cell type changes throughout the ALI-culturing process (Figure 4). Some of these cell type specific gene markers overlapped with the gene clusters identified with gene co-expression analysis. The proliferative gene markers *BIRC5*, *CEP55* and *MKI67* are included in the “chromosome organization” cluster, for the deuterosomal gene markers *CDC20B*, *CEP78* and *PLK4* with the “multi-ciliated epithelial cell differentiation” cluster, and for the multiciliated gene makers *AKAP14* and *SPEF2* with the “microtubule-based movement” cluster. *DNAH5* (multiciliated marker) was not included in the gene clusters identified. Prior to the expression of deuterosomal or multiciliated cell markers, proliferative cell markers have increased expression (maximal on ALI-culture day 4), which subsequently declines throughout the remainder of the ALI culturing process (Figure 4A). The expression of the majority of deuterosomal cell makers appeared on ALI-culture day 4 and peaked on day 14 followed by a progressive decline in expression over the remainder of the culture period (Figure 4B). Finally, the expression of multiciliated cell markers increased, from day 8, reaching peak expression around day 14 to day 21 of culture, followed by a relatively stable expression (Figure 4C).

## Discussion

ALI-culture of nasal epithelial cells enables the differentiation and recapitulation of a pseudostratified epithelium *in vitro*, and can be used to investigate respiratory disease pathogenesis and evaluate therapeutics. In this study, we captured the physiological and global transcriptomic changes, occurring over an extended 63-day ALI-culture period using healthy human nasal epithelial cells, to determine the *in vitro* time-points that best recapitulates the *ex vivo* healthy human nasal brushing cell transcriptome. Whilst it was likely possible to study the ALI-cultures for a longer time period, we chose to sacrifice our ALI-cultures by a maximum cut-off of 2 months to ensure sample health, ciliation and integrity. It was not the purpose of this study to observe when the cell cultures were likely to deteriorate. As a minimum (as per examples: Blume and Jackson et al., 2021 and Bukowy-Bieryłło et al., 2022), three biological samples were used per sub-group for comparative gene expression analysis; the minimum sample size for downstream expression analysis (Conesa et al., 2016; Schurch et al., 2016). Low inter-donor BCV was calculated, also refer to the PCA plot in Figure 2A, suggesting homogeneity between individual donors within sub-groupings; in support of using three samples per sub-grouping. Physiological and transcriptomic results indicated that during the first week of ALI-culture the cells had an increased metabolism and proliferation. From the second week onwards, the cells became increasingly more differentiated as ciliogenesis became widespread. Comparing the transcriptome profiles of the different *in vitro* ALI-culture time-points against the *ex vivo* healthy human nasal brushing cell transcriptome revealed transcriptome similarity from the ALI-culture time-point day 14 onwards.

Prior to this study, others have shown transcriptomic differences between human lower airway epithelial cells cultured at ALI and *ex vivo* bronchoscopy brushing samples (Dvorak et al., 2011); between human nasal epithelial cells at ALI and *ex vivo* nasal brushing samples obtained from former smokers with COPD (Ghosh et al., 2020); and between human tracheal and bronchial epithelial cells cultured at ALI and *ex vivo* tracheal and bronchial brushing samples (Pezzulo et al., 2010). Recently, Bukowy-Bieryłło et al. (2022) characterized primary human nasal epithelial cell differentiation dynamics and inter-donor variability by assessing the expression of fourteen airway epithelium genes (associated with airway epithelium differentiation, specific airway epithelium cell types, and PCD pathogenesis). These authors concluded that the expression of a subset of cilia-related genes is related to the culture time-point, and that inter-individual gene and protein expression changes observed during differentiating airway epithelium cells might reflect the influence of external factors. Despite our two studies using different approaches for functional genomic analysis, we can draw similarities in our culture methods enabling some useful cross-comparisons. Therefore, we consider our study as an extension of the study presented by Bukowy-Bieryłło et al. (2022), giving additional insights into the temporal physiological and whole transcriptomic changes of nasal epithelial cell development and ciliogenesis cultured over an extended ALI-culture period and compared against *ex vivo* brushed cells.

Transcriptomic analysis of *ex vivo* samples and *in vitro* ALI-cultured nasal epithelial cells confirmed previous reports that *ex vivo* samples formed a distinct transcriptome cluster compared to any of the ALI-culture time-points (Pezzulo et al., 2010; Dvorak et al., 2011; Ghosh et al., 2020). The gene expression profile of *in vivo* airway epithelium is therefore not fully represented by cells cultured *in vitro.* As mentioned previously, the transcriptome of the *ex vivo* samples most resembled the transcriptomes of *in vitro* samples from day 14 onwards, yet a major difference was lack of immune response regulation genes *in vitro*, also seen by others (Pezzulo et al., 2010; Dvorak et al., 2011).

Perhaps as expected, the early *in vitro* ALI-cultures (day 1–4) were unpolarized, as shown by TEER (Figure 1D) and microscopically had a “flat” cell appearance (not shown). They also demonstrated a distinct gene cluster compared to later ALI-culture time-points. Heatmap analysis of transcriptomes revealed two major clusters, one associated with the first ALI-culture week (days 1, 4, and 8) and the other associated with the subsequent ALI-culture weeks (days 14, 21, 28, and 63), indicating a large transcriptome dissimilarity over time. The first major cluster contained three sub-clusters corresponding individually to ALI-culture days 1, 4, and 8. GO analysis showed that the most significant gene sub-cluster seen at day 1 consisted of upregulated genes involved with organic acid- and ncRNA metabolic processes which subsequently declined throughout the rest of the ALI culturing process, likely explained by the transfer of cells from the expansion (PneumaCult-Ex Plus) medium to the ALI-culture (Pneumacult-ALI Medium) which contained different metabolic components. Chromosome organization genes were upregulated and peaked at day 4, which would be consistent with DNA replication to initiate ciliogenesis and further cell differentiation. The TEER peak reached at the end of week 1 (Figure 1D) suggested active cell division, which was supported by peak expression of the cell proliferation specific gene markers *BIRC5*, *CEP55*, and *MK167* (Figure 4A) (Ruiz García et al., 2019). Interestingly, Bukowy-Bieryłło et al. (2022) found the expression of *MK167* to decrease during the first weeks of ALI-culture, then stabilizing at a lower level by day 21. While in our study *MKI67,* together with *BIRC5* and *CEP55*, expression markedly increases from ALI-culture day 1 to day 4, followed by a gradual decrease to day 28 and subsequently an apparently stable expression level comparable to day 1 and the *ex vivo* samples. Similarly, Dvorak et al. (2011) and Pezzulo et al. (2010) found that, compared to *ex vivo* brushing samples, expression of genes related to proliferation were increased in respiratory epithelial cells during the first few weeks of ALI-culture. We found an increase in expression of deuterosomal cell gene makers (*CDC20B*, *CEP78*, *PLK4*) (Ruiz García et al., 2019) from ALI-culture day 4 onwards peaking at day 14 (Figure 4). The final differentiation into multiciliated cells is initiated by *GEMC1* and *MCIDAS* (geminin family genes), that activate transcription factors *p73* and *FOXJ1* instigating deuterosome manufacture from parental centrioles. These structures then act as platforms for centriole amplification, scaled to the cell surface area, before they are translocated and docked at the apical cell membrane to initiate basal body formation. There is also a contention that centrioles can be formed *de novo* from pericentriolar material and fibrogranular material near the nuclear membrane (Rayamajhi and Roy, 2020). Multi-ciliated epithelial cell differentiation genes were expressed at their highest at day 8 (Figure 3) and specific multiciliated gene markers (Revinski et al., 2018; Ruiz García et al., 2019) were highly upregulated from day 8 onwards and peaked between day 14 and day 28 (Figure 4C). Accordingly, genes involved with ciliary transition zone assembly, microtubule-based movement and cilium organization started to be expressed between day 4 and day 8 of ALI-culture and interestingly did not peak, plateau or decline, but increased up to our latest time-point of day 63 (Figures 2, 3). Previously, Pezzulo et al. (2010) reported that expression of genes associated with cilia structure and function were upregulated in *ex vivo* brushing samples compared to ALI-cultures harvested at 2 weeks. However, in our study cilia-related gene clusters and multiciliated cell gene markers were at comparable expression levels between ALI-culture day 14. Both the Pezzulo et al. (2010) and this study compared like-for-like. Pezzulo et al. (2010) compared brushing- and ALI-culture samples derived from tracheal and bronchial cells against each other, and in this study brushing- and ALI-culture samples derived from nasal epithelial cells were compared against each other. Hence why the *ex vivo* and *in vivo* differences in cilia structure and function related gene expression might be due to differences in experimental set-up.

Cilia were microscopically observed by day 7 in ALI-culture using HSVMA and cilia coverage increased from 3% (day 7) to 38% (day 29) (Figures 1A,B). So, the early increases in gene expression around ALI-culture days 1 to 8 are likely related to active cell differentiation, and ciliation itself happens rapidly between days 4 and 8 when cilia related genes become expressed. Furthermore, by weekly *in situ* measurements at 37°C we determined that mean CBF remained between 7.1 and 9.4 Hz from day 7 to day 63 (Figure 1B). Despite weekly washing of the apical ALI-culture surface, mucus build-up *in situ* contributed to a slight reduction in CBF compared to that measured after culture scraping and extra washing in Coles et al. (2020). One ALI-culture was immeasurable at day 63 due to mucus build-up suggesting mucus can be a problem and an apical surface washing regime might be necessary. Thus, for a better assessment of CBF, we would advise harvesting the cells by scraping as described (Coles et al., 2020).

The second major gene cluster contained three sub-clusters belonging to the transcriptomes of the *ex vivo* samples, the *in vitro* ALI-cultures from days 14 and 21, the *in vitro* ALI-cultures from days 28 and 63. Genes related to positive regulation of RNA metabolic processes slowly increased in ALI-culture to day 14/21 and expression appears to decline for the remainder of the ALI-culture. As mentioned earlier genes involved with ciliary transition zone assembly, micro-tubule-based movement and cilium organization were associated with the latest ALI-culture time-points, while expression started between day 4 and day 8. At day 28 the ALI-cultured epithelial cells were deemed fully differentiated, with presence of the tight junction marker E-cadherin (immunofluorescent labelling), a polarized epithelial barrier, widespread dense ciliation (seen by SEM and α-tubulin immunofluorescence-labelling) estimated to 38% coverage, goblet cells (MUC5AC intracellular expression increased from day 14 to day 28) and mucus production (observed during culture surface washes) (Figure 1).

In conclusion, although *ex vivo* nasal brushing samples formed distinct transcriptome clusters to *in vitro* ALI-cultured nasal epithelia, day 14 was the earliest time-point that best matched the *ex vivo* samples. However, immune response regulation genes were deficient in the *in vitro* ALI-culture samples compared to the *ex vivo* nasal brushing samples, likely because the *in vitro* cultures lack an airway microbiome, lack stimulation by airborne particles, and/or did not host an immune cell component. This highlights the need for more advanced co-cultures with immune cell representation to better reflect the physiological state. Epithelial cell barrier function plateaus from the end of week 1 and ciliation can occur within 7 days of *in vitro* ALI-culture, although widespread ciliation is not complete until day 28, therefore harvesting time-points need to be considered to suit the purpose of investigation (transcriptomic and/or functional analysis).

## Data Availability

The datasets presented in this study can be found in online repositories. The names of the repository/repositories and accession number(s) can be found below: https://www.ncbi.nlm.nih.gov/sra, PRJNA650028.
