# Supplementary material for: Temporal Whole-Transcriptomic Analysis of Characterized In Vitro and Ex Vivo Primary Nasal Epithelia
Source: Front Cell Dev Biol. 2022 Jun 15;10:907511. doi: 10.3389/fcell.2022.907511 (PMC9240208; doi:10.3389/fcell.2022.907511)
Supplement: Supplementary file 1 [file Table1.docx]

**FastQC, Picard, RSeQC and MultiQC**

## FastQC

sample1_1 = ./$sample'_1.fq.gz'

sample1_2 = ./$sample'_2.fq.gz'

fastqc $sample1_1 $sample1_2 --outdir = ./analysed/$project/fastqc/$sample

## Picard CollectRNAseqMetrics

$java -jar $picard_jar CollectRnaSeqMetrics \

I = ./$project/Picard/$sample /$sample.sorted.bam \

O = ./$project/Picard/$sample/$sample.rnaseq_metrics \

CHART = . /$project/Picard/$sample/$sample.rnaseq.pdf \

REF_FLAT = . /resources/refFlat.txt \

STRAND = SECOND_READ_TRANSCRIPTION_STRAND \

RIBOSOMAL_INTERVALS = ./resources/CHRadjusted.gencode.GRCh38.v35.rRNA.interval_list

## Picard CollectInsertSizeMetrics

$java -jar $picard_jar CollectInsertSizeMetrics \

I = ./$project/alignment/$sample/$sample.sorted.bam \

O = ./$project/Picard/$sample/$sample.insert_size_metrics \

H = ./$project/Picard/$sample/$sample.insert_size_metrics.pdf \

M = 0.5

## Genebody coverage

./.local/bin/geneBody_coverage.py \

-i ./$project/alignment/$sample/$sample.sorted.bam \

-r ./resources/hg38_Gencode_v35.bed \

-o ./$project/Picard/$sample

## Junction saturation

./.local/bin/junction_saturation.py

-i ./$project/alignment/$sample/$sample.sorted.bam

-o ./$project/Picard/$sample

-r ./resources/hg38_Gencode_v35.bed

## Junction annotation

./.local/bin/junction_annotation.py

-i ./$project/alignment/$sample/$sample.sorted.bam

-o ./$project/Picard/$sample

-r ./resources/hg38_Gencode_v35.bed

## MultiQC

multiqc ./[input folder e.g. alignment of a sample] -o ./MultiQC/[alignment sample]

**STAR**

## STAR genome generate step

STAR \

--runMode genomeGenerate \

--genomeDir . /resources/GTex_STAR_b38_v35_genome/ \

--genomeFastaFiles ./resources/GRCh38.primary_assembly.genome.fa \

--runThreadN 16 \

--sjdbGTFfile ./resources/gencode.v35.annotation.gtf \

--sjdbOverhang 149

## STAR Alignment

sample1_1 = ./$sample'_1.fq.gz'

sample1_2 = ./$sample'_2.fq.gz'

./tools/STAR-2.7.3a/bin/Linux_x86_64_static/STAR \

--genomeDir ./resources/GTex_STAR_b38_v35_genome/ \

--readFilesCommand zcat \

--readFilesIn $sample1_1 $sample1_2 \

--runThreadN 16 \

--twopassMode Basic \

--twopass1readsN -1 \

--outSAMmapqUnique 60 \

--outFilterType BySJout \

--outFilterMultimapNmax 20 \

--alignSJoverhangMin 8 \

--alignSJDBoverhangMin 3 \

--outFilterMismatchNmax 999 \

--outFilterMismatchNoverReadLmax 0.04 \

--alignIntronMin 20 \

--alignIntronMax 1000000 \

--limitSjdbInsertNsj 2000000 \

--alignMatesGapMax 1000000 \

--quantMode GeneCounts \

--outReadsUnmapped Fastx \

--outSAMtype BAM Unsorted

## SamTools script

samtools sort ./Aligned.out.bam > ./$sample.sorted.bam

samtools index ./$sample.sorted.bam

**HTSeq**

## HTSeq script

BAMfile = ./$project/alignment/$sample/$sample.sorted.bam

GTF = ./resources/gencode.v35.annotation.gtf

./HTSEQ/bin/htseq-count --format=bam --order=pos --stranded=reverse --max-reads-in-buffer=150000000 --mode=union --a=1 --type=exon $BAMfile $GTF > ./$project/HTSeq/$sample/$sample.rawReadCounts.txt

**edgeR (PCA plot and heatmap)**

# Load libraries

library(edgeR)

library(Biobase)

library(ggplot2)

library(heatmap.plus)

library(RColorBrewer)

library(tidyverse)

library(ggrepel)

# Filter out low count genes and TMM normalisation

# Place raw data in variable

setwd(paste("C:/Bureaublad/PHD/Gene expression/Timepoints/all_times"))

rawData <- read.table("./all_times.finalAllRawReadCounts.txt", header=T, row.names=1, sep="\t")

# Define the experimental groups

# expGroups <- factor( c( rep("X",3), rep("Y",3) ))

expGroups <- factor( c( rep("00",3), rep("01",3), rep("04",3), rep("08",3), rep("14",3), rep("21",3), rep("28",3), rep("63",3) ))

# Filtering raw data

keep <- filterByExpr(rawData, group=expGroups)

filteredData <- rawData[keep, ]

# Make an edgeR object to store all the DGE info

edgeRData <- DGEList(counts=filteredData, genes=rownames(filteredData), group=expGroups)

# Make a bar plot of the number of reads, ie library size, in each sample

barplot(edgeRData$samples$lib.size,names=colnames(edgeRData),las=2,xaxt="n")

title("Barplot of library sizes")

# Calculate counts per million per sample

# Count to be added to each observation to avoid taking log of zero.

logCPM <- cpm(edgeRData,log=TRUE,lib.size=libsize,prior.count=1)

# Check distributions of samples using boxplots

# Add a title and blue horizontal line corresponding to the median logCPM

boxplot(logCPM, xlab="", ylab="Counts per million (Log)",las=2,xaxt="n")

abline(h=median(logCPM),col="blue")

title("Boxplots of logCPMs (TMM unnormalised)")

# Normalise the filtered raw counts using the TMM method

edgeRData <- calcNormFactors(edgeRData, method="TMM")

# Calculate counts per million per sample

# Count to be added to each observation to avoid taking log of zero.

CPM <- cpm(edgeRData,log=FALSE, lib.size=libsize,prior.count=1)

logCPM <- cpm(edgeRData,log=TRUE,lib.size=libsize,prior.count=1)

# Check distributions of samples using boxplots

# Add a title and blue horizontal line corresponding to the median logCPM

boxplot(logCPM, xlab="", ylab="Counts per million (Log)",las=2,xaxt="n")

abline(h=median(logCPM),col="blue")

title("Boxplots of logCPMs (TMM normalised)")

# Make MDS plot

Legend <- c("RNA-later","ALI day 1","ALI day 4","ALI day 8","ALI day 14","ALI day 21","ALI day 28","ALI day 63")

col.expGroups <- c("red","blue","green","yellow","black","purple","brown","orange")[expGroups]

plotMDS(edgeRData, col=col.expGroups, cex=0.5,method="logFC")

legend("bottomleft",fill=c("red","blue","green","yellow","black","purple","brown","orange"),legend=Legend)

title("MDS plot")

# Save results

write.table(CPM, file="TMM_CPM_corrected.txt", quote=FALSE, row.names=TRUE, sep="\t")

# PCA plot

pca <- prcomp(t(CPM), scale=TRUE)

# Calculate percentage

pca.var <- pca$sdev^2

pca.var.per<-round(pca.var/sum(pca.var)*100, 1)

barplot(pca.var.per,main="Scree Plot all genes", xlab="Principal Component",ylab="Percent Variation", ylim=c(0,35))

# Make PCA data frame

pca.data <- data.frame(Sample=rownames(pca$x), X=pca$x[,1], Y=pca$x[,2])

Timepoint <- c(rep("Ex vivo (RNA-later)", 3),rep("ALI (day 01)",3),rep("ALI (day 04)",3),rep("ALI (day 08)",3),rep("ALI (day 14)",3),rep("ALI (day 21)",3),rep("ALI (day 28)",3),rep("ALI (day 63)",3))

pca.data$Timepoint<-Timepoint

# Make PCA plot

ggplot(data=pca.data, aes(x=X, y=Y, label=Sample, shape=Timepoint)) +

geom_vline(xintercept = c(0), linetype="dashed", col="darkgrey") +

geom_hline(yintercept = c(0), linetype="dashed", col="darkgrey") +

geom_point(size=2) +

scale_shape_manual(values=c(0,1,2,3,4,9,6,7,8)) +

xlab(paste("PC1 - ", pca.var.per[1], "%", sep="")) +

ylab(paste("PC2 - ", pca.var.per[2], "%", sep="")) +

theme_bw() +

ggtitle("") +

theme(plot.title = element_text(hjust = 0.5)) +

theme(legend.title=element_blank())

# Heatmap

# Set the timepoint annotation for each sample

annotation_col1 = data.frame(Source=c(rep("RNA-later", 3),rep("ALI",21)))

annotation_col2 = data.frame(Timepoint=c(rep("Day 00", 3),rep("Day 01",3),rep("Day 04",3),rep("Day 08",3),rep("Day 14",3),rep("Day 21",3),rep("Day 28",3),rep("Day 63",3)))

annotation_col <- cbind(annotation_col1, annotation_col2)

rownames(annotation_col) <- colnames(CPM)

annotation_colours <- list(Source=c('RNA-later'="#FDE725FF", ALI="#440154FF"), Timepoint=c("Day 00"="#440154FF", "Day 01"="#46337EFF", "Day 04"="#365C8DFF", "Day 08"="#277F8EFF", "Day 14"="#1FA187FF", "Day 21"="#4AC16DFF", "Day 28"="#9FDA3AFF", "Day 63"="#FDE725FF"))

pheatmap(CPM,

color=viridis::plasma(25),

show_rownames=FALSE,

show_colnames=FALSE,

annotation_col=annotation_col,

annotation_colors=annotation_colours,

scale="row",

clustering_method="ward.D2",

clustering_distance_cols="euclidean",

cutree_cols=5,

cutree_rows=5)

**edgeR (differential gene expression)**

# Define the experimental groups

# expGroups <- factor( c( rep("X",3), rep("Y",3) ))

expGroups <- factor( c( rep("BRUSH",3), rep("ALI",21)))

# Filtering raw data

keep <- filterByExpr(rawData, group=expGroups)

filteredData <- rawData[keep, ]

# Make an edgeR object to store all the DGE info

edgeRData <- DGEList(counts=filteredData, genes=rownames(filteredData), group=expGroups)

# Make a bar plot of the number of reads, ie library size, in each sample

barplot(edgeRData$samples$lib.size,names=colnames(edgeRData),las=2,xaxt="n")

title("Barplot of library sizes")

# Calculate counts per million per sample

# # Count to be added to each observation to avoid taking log of zero.

logCPM <- cpm(edgeRData,log=TRUE,lib.size=libsize,prior.count=1)

# Check distributions of samples using boxplots

# Add a title and blue horizontal line corresponding to the median logCPM

boxplot(logCPM, xlab="", ylab="Counts per million (Log)",las=2,xaxt="n")

abline(h=median(logCPM),col="blue")

title("Boxplots of logCPMs (TMM unnormalised)")

# Normalise the filtered raw counts using the TMM method

edgeRData <- calcNormFactors(edgeRData, method="TMM")

# Calculate counts per million per sample

# Count to be added to each observation to avoid taking log of zero.

logCPM <- cpm(edgeRData,log=TRUE,lib.size=libsize,prior.count=1)

# Check distributions of samples using boxplots

# Add a title and blue horizontal line corresponding to the median logCPM

boxplot(logCPM, xlab="", ylab="Counts per million (Log)",las=2,xaxt="n")

abline(h=median(logCPM),col="blue")

title("Boxplots of logCPMs (TMM normalised)")

# Make MDS plot

Legend <- c("BRUSH","ALI")

col.expGroups <- c("red","blue","green","yellow","black","purple","brown","orange")[expGroups]

plotMDS(edgeRData, col=col.expGroups, cex=0.5,method="logFC")

legend("bottomleft",fill=c("red","blue","green","yellow","black","purple","brown","orange"),legend=Legend)

title("MDS plot")

# Estimate dispersion

edgeRData <- estimateCommonDisp(edgeRData, verbose=TRUE)

edgeRData <- estimateTrendedDisp(edgeRData)

edgeRData <- estimateTagwiseDisp(edgeRData)

# Make BCV plot

plotBCV(edgeRData, xlab="Average log CPM", ylab="Biological coefficient of variation", pch=14, cex=0.2, col.common="red", col.trend="blue",mcol.tagwise="black")

title("Biological coefficient of variation (BCV) against gene abundance (in log2 CPM)")

# Identify differentially expressed genes using an exact test

# Print the most significant genes

dge <- exactTest(edgeRData)

topTags(dge)

# Print number of up/down significant genes at FDR = 0.05 significance level

summary(sig_dge <- decideTestsDGE(dge, p=.05))

sig_dgeTags <- rownames(edgeRData)[as.logical(sig_dge)]

# Make a smear plot

plotSmear(dge, de.tag=sig_dgeTags, xlab="Average logCPM", ylab="logFC")

title("Smearplot")

# Start preparing results output file by putting selected variables into one data frame (table) for all genes tested for DGE

dgeResults <- data.frame(rownames(dge), dge$table$PValue, log10(dge$table$PValue), p.adjust(dge$table$PValue, "fdr"), dge$table$logFC, dge$table$logCPM)

# Add column names to the results

colnames(dgeResults) <- c("HGNC_ENSG", "PValue", "Log10_PValue", "FDR_PValue", "Log_fold_change", "Log_CPM")

# Make a variable that will be used to sort the results from most to least significant gene

# and use the sort variable to sort the results

sortOrder <- order(dge$table$PValue,decreasing=FALSE)

dgeResults <- dgeResults[sortOrder,]

# Calculate logCPMs per sample and sort by PValue, most significant first

logCPM_Heatmap <- cpm(edgeRData[sortOrder[1:length(sortOrder)],],log=TRUE, lib.size=libsize,prior.count=1)

# Add counts per million per sample to results

all_results <- data.frame(dgeResults,logCPM_Heatmap)

# Save the results to a file called dge_results.txt

write.table(all_results, file="dge_results_BRUSH_vs_ALI.txt", quote=FALSE, row.names=FALSE, sep="\t")

# Volcano plot

dge_results <- all_results

# The significantly differentially expressed genes are the ones found in the upper-left and upper-right corners.

# Add a column to the data frame to specify if they are UP- or DOWN- regulated (log2FoldChange respectively positive or negative)

# add a column of NAs

dge_results$diffexpressed <- "Not Sig"

# if log2Foldchange > 1 and pvalue < 0.05, set as "UP"

dge_results$diffexpressed[dge_results$Log_fold_change > 1 & dge_results$FDR_PValue < 0.05] <- "Up"

# if log2Foldchange < -1 and pvalue < 0.05, set as "DOWN"

dge_results$diffexpressed[dge_results$Log_fold_change < -1 & dge_results$FDR_PValue < 0.05] <- "Down"

# Change point color

DE_colors <- c("blue", "red", "grey")

names(DE_colors) <- c("Down", "Up", "Not Sig")

# Make volcano plot

ggplot(dge_results, aes(x=Log_fold_change,y=-log10(FDR_PValue),colour= diffexpressed)) +

scale_colour_manual(values=DE_colors) +

geom_point(size = 1) +

geom_vline(xintercept = c(-1,1), linetype="dashed", col="black") +

geom_hline(yintercept = -log10(0.05), linetype="dashed", col="black") + geom_text_repel(aes(label=ifelse(Gene=="LCP1"|Gene=="C1QC"|Gene=="PTPRC"|Gene=="DMBT1"|Gene=="FGL2"|Gene=="MS4A6A"|Gene=="C1QA"|Gene=="MPEG1"|Gene=="C1QB"|Gene=="SPN", as.character(Gene),'')), size=3, max.overlaps=Inf) +

ylab("-log10(FDR)") +

xlab("log2(fold change)") +

theme_classic() +

theme(plot.title = element_text(hjust = 0.5)) +

theme(legend.title=element_blank())
